# SGSNet: a lightweight deep learning model for strawberry growth stage detection

**DOI:** 10.3389/fpls.2024.1491706

**Published:** 2024-12-02

**Authors:** Zhiyu Li, Jianping Wang, Guohong Gao, Yufeng Lei, Chenping Zhao, Yan Wang, Haofan Bai, Yuqing Liu, Xiaojuan Guo, Qian Li

**Affiliations:** School of Computer Science and Technology, Henan Institute of Science and Technology, Xinxiang, China

**Keywords:** deep learning, strawberry growth stages detection, lightweight, SGSNet, GrowthNet, DySample

## Abstract

**Introduction:**

Detecting strawberry growth stages is crucial for optimizing production management. Precise monitoring enables farmers to adjust management strategies based on the specific growth needs of strawberries, thereby improving yield and quality. However, dense planting patterns and complex environments within greenhouses present challenges for accurately detecting growth stages. Traditional methods that rely on large-scale equipment are impractical in confined spaces. Thus, the development of lightweight detection technologies suitable for portable devices has become essential.

**Methods:**

This paper presents SGSNet, a lightweight deep learning model designed for the fast and accurate detection of various strawberry growth stages. A comprehensive dataset covering the entire strawberry growth cycle is constructed to serve as the foundation for model training and testing. An innovative lightweight convolutional neural network, named GrowthNet, is designed as the backbone of SGSNet, facilitating efficient feature extraction while significantly reducing model parameters and computational complexity. The DySample adaptive upsampling structure is employed to dynamically adjust sampling point locations, thereby enhancing the detection capability for objects at different scales. The RepNCSPELAN4 module is optimized with the iRMB lightweight attention mechanism to achieve efficient multi-scale feature fusion, significantly improving the accuracy of detecting small targets from long-distance images. Finally, the Inner-IoU optimization loss function is applied to accelerate model convergence and enhance detection accuracy.

**Results:**

Testing results indicate that SGSNet performs exceptionally well across key metrics, achieving 98.83% precision, 99.45% recall, 99.14% F1 score, 99.50% mAP@0.5, and a loss value of 0.3534. It surpasses popular models such as Faster R-CNN, YOLOv10, and RT-DETR. Furthermore, SGSNet has a computational cost of only 14.7 GFLOPs and a parameter count as low as 5.86 million, demonstrating an effective balance between high performance and resource efficiency.

**Discussion:**

Lightweight deep learning model SGSNet not only exceeds the mainstream model in detection accuracy, but also greatly reduces the need for computing resources and is suitable for portable devices. In the future, the model can be extended to detect the growth stage of other crops, further advancing smart agricultural management.

## 1 Introduction

Strawberries are highly valued for their rich nutrients and unique flavor (Xiao et al., 2020). The ripeness of strawberries directly determines the optimal harvest time (Wang et al., 2024f). Harvesting too early results in adequate nutrition and good taste, while harvesting too late leads to spoilage, affecting sales (Ma et al., 2024). Determining the growth stages of strawberries primarily relies on manual inspection, which is highly subjective, inconsistent, labor-intensive, and prone to causing damage to the fruit during observation (Chen et al., 2022). Using computer vision technology (Wang et al., 2023d) to detect the growth stages of strawberries ensures accuracy, helps farmers accurately predict the optimal harvest time (Pan et al., 2024), enables timely picking, prevents waste caused by delayed harvesting, and reduces physical damage from manual inspection, thereby improving the quality and flavor of strawberry products (Tang et al., 2023b).

Despite advancements in computer vision technology, existing deep learning models for detecting strawberry growth stages continue to encounter numerous challenges. First, most deep learning models, such as Faster R-CNN and Mask R-CNN (Mahmood et al., 2022), are designed with a large number of parameters, leading to high computational complexity that limits their applicability on lightweight devices like smartphones and drones. Second, You Only Look Once (YOLO) models and their variants are widely employed for real-time applications. While these models are optimized for speed, this often compromises detection accuracy, making it difficult to accurately identify small objects or those with complex features (Ye et al., 2023). In particular, the direct application of YOLO models for detecting strawberry growth stages presents significant challenges (Zhang et al., 2024a). Additionally, factors such as uneven lighting and dense plant growth in greenhouses complicate the environment, further increasing the difficulty of detection at each growth stage of strawberries. Therefore, developing a lightweight deep learning model (Wang et al., 2023a) designed explicitly for detecting strawberry growth stages is crucial.

This paper presents SGSNet, a novel lightweight deep learning model designed to quickly and accurately detect the various growth stages of strawberries. The main contributions are as follows:

(1) We have constructed a comprehensive dataset covering all stages of strawberry growth, providing a versatile sample set tailored for training and testing deep learning models.
(2) We designed GrowthNet as the backbone of SGSNet, a lightweight convolutional neural network that efficiently extracts data features while minimizing model parameters and complexity. SGSNet also integrates the DySample adaptive upsampling structure, which dynamically adjusts sampling points to enhance detection across multiple scales.
(3) We optimized the RepNCSPELAN4 module with the lightweight iRMB attention mechanism, enabling efficient multi-scale feature fusion and significantly enhancing the accuracy of small object detection in long distance captures.
(4) We enhanced the loss function with Inner-IoU to improve bounding box regression. This approach significantly accelerates convergence and boosts detection accuracy, especially when there is a large discrepancy between predicted and ground truth boxes.

The rest of the paper is organized as follows: Section 2 discusses related work; Section 3 introduces the materials and methods; Section 4 conducts experimental analysis; Section 5 is the discussion; and finally, Section 6 summarizes this article.

## 2 Related work

The application of computer vision technology in the agricultural sector has been increasingly widespread. Li et al. (2024). proposed a lightweight and highly accurate pitaya fruit detection model by improving the YOLOv5s architecture, incorporating the ShuffleNetV2, C3RFE module, BiFPN feature fusion, and SE attention mechanisms, successfully deploying the model on Android devices. Evarist et al. (2024). developed a convolutional neural network-based model that leverages image processing and transfer learning techniques to achieve high-precision detection of pesticide residues in the edible parts of vegetables such as tomatoes, cabbages, carrots, and green peppers, with the Inception V3 model achieving the highest accuracy at 96.77%. Computer vision technology provides an automated and efficient method for detecting strawberry growth stages, significantly reducing reliance on human labor, speeding up the detection process, and effectively minimizing the number of under-ripe or overripe strawberries entering the market. This technology also facilitates the sorting and harvesting of strawberries, ensuring they are delivered to consumers in high-quality batches, enhancing their commercial value. Notable progress has been made in this field. Xu et al. (2013). Histogram of Oriented Gradients (HOG) descriptors are combined with a Support Vector Machine (SVM), and the HOG descriptors are applied to the classifier to achieve accurate strawberry detection.

However, traditional computer vision techniques require manual feature design, which needs to be improved to meet the real-time detection needs of strawberries at various growth stages. To address this limitation, some studies have proposed optimizing computer vision techniques for real-time strawberry detection using deep learning. Chen et al. (2019). developed an automated strawberry flower detection system using the Faster R-CNN network and transfer learning from ImageNet, which accurately detects strawberry flowers to predict yield. Wang et al. (2023c). proposed an ASFA-net-based method that classifies strawberry ripeness using the proportion of red pixels while achieving high-precision localization and detection. Tang et al. (2023a). introduced a method based on Mask R-CNN and image processing techniques to achieve fine maturity recognition of strawberries in the field, supporting precision farming management. Liu et al. (2024a). developed a novel task-aligned single-stage object detection method, offering a new approach for detecting strawberries with complex shapes and classifying their maturity. Zhu et al. (2024) proposed an unsupervised deep learning-based method for detecting the external quality of strawberries, addressing the limitations of supervised learning that relies on prior knowledge to segment datasets. Soode-Schimonsky et al. (2017) applied a product environmental footprint approach to compare and analyze various strawberry farming systems in Germany and Estonia. Anjom et al. (2018) developed an intelligent harvester integrated with load sensors, RTK GPS, a microprocessor, and an inertial measurement unit, which measures strawberry yield in blocks. Gao et al. (2020) estimated strawberry ripeness in field and laboratory conditions using a hyperspectral imaging (HSI) system. Yu et al. (2019). utilized Mask R-CNN, for instance, in the segmentation of strawberries, outperforming traditional computer vision methods in scenarios with complex backgrounds and varying light conditions and cases of fruit overlap and occlusion.

As the efficiency and precision of You Only Look Once (YOLO) series single-stage object detection models become increasingly prominent; more researchers have enhanced YOLO models for detecting various growth stages of strawberries. Zhang et al. (2022). developed a strawberry detection algorithm based on YOLOv4-tiny, enabling real-time detection of strawberry fruits. Wang et al. (2022). proposed the DSE-YOLO model for multi-stage strawberry fruit detection, addressing challenges such as small strawberry size, foreground-background imbalance, and complex natural environments. Li et al. (2022b). introduced a computer vision-based algorithm for detecting PM and IL in strawberry leaves and improved the original YOLOv4 model by incorporating deep convolution and hybrid attention mechanisms. Yang et al. (2023). proposed a strawberry maturity detection model based on the YOLOv8 algorithm integrated with the LW-Swin Transformer, utilizing the Transformer’s multi-head self-attention mechanism to capture long-range dependencies in the input data. Wang et al. (2024c). developed a method combining deep learning with image processing to identify and classify strawberry maturity, enhancing the YOLOv8 model with an ECA attention mechanism and Focal-EIOU loss function to improve recognition performance. Du et al. (2023). developed an improved DSW-YOLO network model for strawberry fruit recognition and occlusion detection. Zhou et al. (2021). applied the YOLOv3 deep learning method to classify strawberry growth stages using aerial and ground images, demonstrating that YOLOv3 can effectively classify growth stages regardless of image type. Yu et al. (2020). proposed the R-YOLO model, which uses the lightweight MobileNet-V1 as the backbone network for feature extraction, improving the accuracy of locating picking points for trench-grown strawberries. Constante et al. (2016). employed a noise-compensated learning strategy to train a robust network-object relationship model, enabling the recognition of complex strawberry features under varying lighting conditions, sizes, and orientations. An et al. (2022). developed the ZDNet model for strawberry growth stage detection, based on the YOLOX model, to improve the accuracy of small fruit detection and attention weighting.

In summary, although some progress has been made in detecting strawberry growth stages, certain limitations remain.

(1) Deep learning techniques have introduced innovative methods for detecting strawberry growth stages without the need for manual feature design. However, the effective training of these models typically relies on large-scale datasets that encompass the entire growth cycle and include precise annotations. Research indicates that acquiring such comprehensive and high-quality datasets is challenging, which limits the training efficiency and overall performance of deep learning models.
(2) Research on detecting strawberry growth stages predominantly focuses on mature fruits, often neglecting the entire growth cycle from planting to maturity. This oversight hinders accurate predictions of fruit ripening times. Significant technical challenges persist in detecting strawberries of varying sizes, particularly in accurately distinguishing between the fruit expansion and color-turning stages.
(3) Most existing models for detecting strawberry growth stages encounter challenges associated with large parameter sizes and high computational costs. Since strawberry growth stage detection must occur in real-time within complex and variable greenhouse environments to monitor the maturity of numerous strawberries, these models must be deployable on lightweight devices. Consequently, developing lightweight deep learning models is essential for overcoming this bottleneck.

## 3 Materials and methods

### 3.1 Data sources

A dataset comprising images of strawberries at various growth stages is utilized, collected from multiple strawberry plantations in Xinxiang City, Henan Province, China (longitude: 113.9202062, latitude: 35.3021133). Each plantation covers an area exceeding 5,000 square meters. The region, characterized by flat terrain, has well-drained and aerated soil, efficiently improved through cultivation and fertilization, providing an optimal environment for strawberry growth.

**Figure 1:**
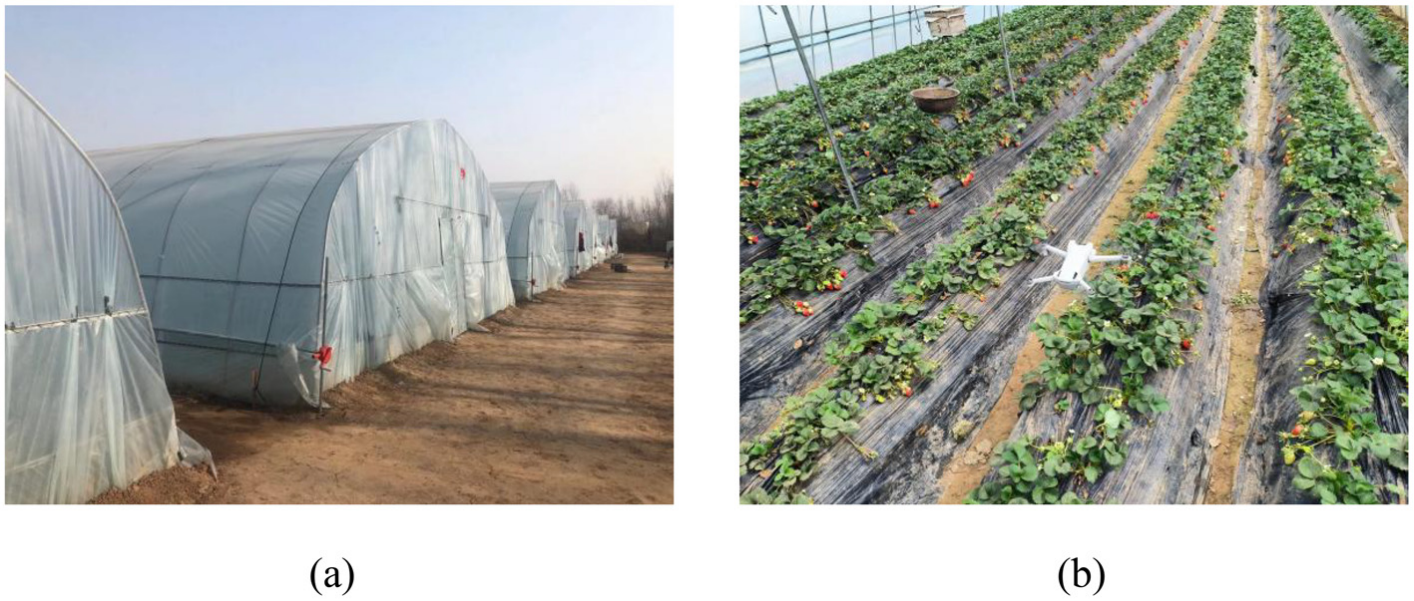
Data collection scenarios. **(A)** shows the greenhouse where different strawberry varieties are cultivated; **(B)** depicts the drone capturing data within the strawberry greenhouse.

The experiment primarily focuses on the Zhongmei Series, Toyonoka, and Sweet Charlie, cultivated in greenhouses across several strawberry plantations. These varieties exhibit consistent morphology throughout their growth stages. Data collection is primarily conducted through manual photography. Considering the dense planting in some regions of the greenhouses, high-resolution drone aerial imaging is employed as an auxiliary method to prevent damage to ripening strawberries; the strawberry greenhouse and the data collection method are shown in **Figure 1**.

Specifically, high-resolution images of strawberries at various growth stages are captured within local greenhouse strawberry orchards using professional-grade equipment, including a Canon camera (Canon Inc., Chaoyang District, Beijing, China) and a DJIMini3 drone (DJI Innovations, Nanshan District, Shenzhen, Guangdong Province, China).

**Figure 2:**
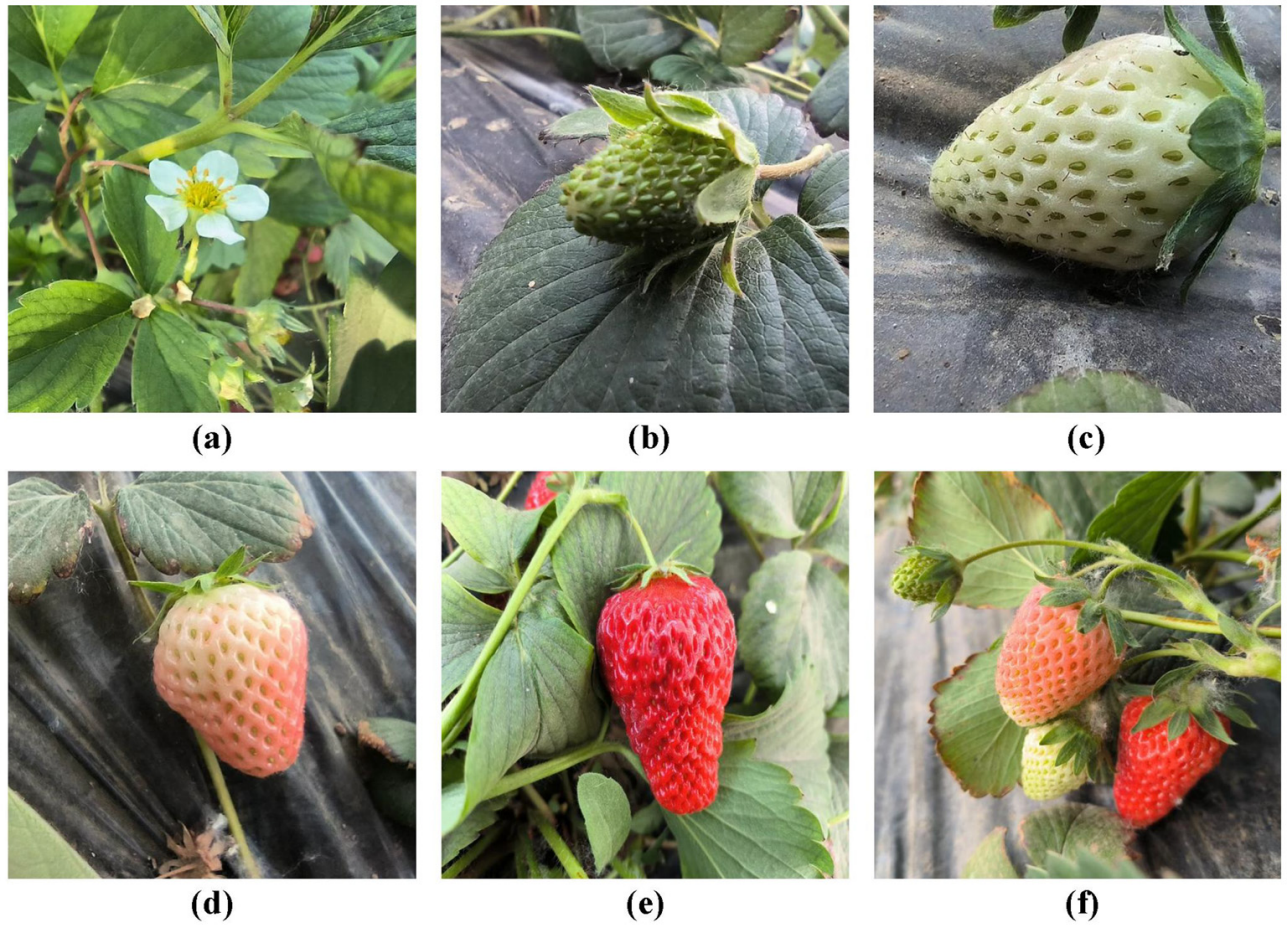
Different growth stages of strawberries. **(A)** Flowering; **(B)** Young Fruit; **(C)** Fruit Expansion; **(D)** Color Turning; **(E)** Maturation; **(F)** Multi-Stage Images.

The strawberry growth stage dataset includes five distinct growth stages: Flowering, Young Fruit, Fruit Expansion, Color Turning, and Maturation. To enhance the training performance of deep learning models on the strawberry growth stage dataset, multiple images capturing strawberries at various growth stages are taken and collectively labeled as Multi-Stages, as shown in **Figure 2**.

The characteristics of strawberries at different growth stages exhibit significant variations, detailed in **Table 1**.

**Table 1:** Detailed description of the characteristics of different growth stages of strawberries.

Growth Stage	Feature Description
Flowering	At this stage, the strawberry plant begins to flower. The petals are white with a yellow center, and the calyx opens.
Young Fruit	The strawberry begins to form young fruit, which is very small and green. The seeds on the surface become more pronounced and closely packed.
Fruit Expansion	As the fruit enters the expansion stage, it increases in size, and its color gradually changes from green to pale white. Both volume and weight increase significantly.
Color Turning	The fruit changes color, transitioning from light white to pink or red. The seeds become more visible, and the color of the calyx changes from green to light yellow.
Maturation	The fruit becomes plump, and its color becomes vibrant red or deep red. The calyx color varies from deep to light, with some parts turning brown.

### 3.2 Data processing methods

In this paper, advanced data processing techniques are employed to optimize the quality of datasets across various stages of the strawberry growth cycle (Zhang et al., 2024b). Given the limitations in sample collection during different phases of strawberry production and the scarcity of corresponding public data resources (Wang et al., 2024g), this research innovatively applies data augmentation techniques to expand the scale and diversity of the existing dataset effectively. Specifically, the data augmentation process is implemented through image flipping and mirroring. Where image flipping includes both horizontal and vertical methods.

We enhanced images under varying lighting conditions by adjusting brightness, darkness, and chroma, which not only augmented the original dataset but also improved image quality, making the data more suitable for deep learning model training. Brightness adjustment techniques effectively simulate lighting variations, reduce noise, and optimize overall image quality. The pixel value after brightness adjustment is represented as *I_Brightness_* (*x,y,z*).


(1) $$I_{Brightness}\left(x,y,z\right)=I(x,y,z)+S_{Brightness}$$

where *I(x,y,z)* represents the pixel value of the original image, with (*x,y,z*) denoting the pixel coordinates, where *z* represents the color channel, *S_Brightness_* indicates the intensity of the brightness adjustment, which is added to each pixel value to brighten the image.

Darkness adjustment is the inverse operation of brightness adjustment, reducing the overall brightness of the image. The pixel value after darkness adjustment is represented as *I_Darkness_* (*x,y,z*), as shown in Equation 2.


(2) $$I_{\text{Darkness}}(x,y,z)=I(x,y,z)-S_{\text{darkness}}$$

where *S_Darkness_* represents the intensity of the darkness adjustment, which is subtracted from each pixel value to darken the image.

Chroma adjustment can enhance or reduce the color saturation of an image, allowing it to adapt to varying environmental conditions and improving image quality by making features at different growth stages more distinct. This process involves adjusting the color components of each pixel, with the adjusted pixel values represented as *I_Chroma_* (*x,y,z*), as shown in Equation 3.


(3) $$I_{Chroma}\left(x,y,z\right)=I(x,y,z)+S_{Chroma}\times \left(I(x,y,z)-G(I(x,y))\right)$$

where *S_Chroma_* represents the intensity of the chroma adjustment, while *G*(*I*(*x,y*)) denotes the average value of the color channels at a specific position in the original image, used to minimize the impact of noise or fine details.

**Figure 3:**
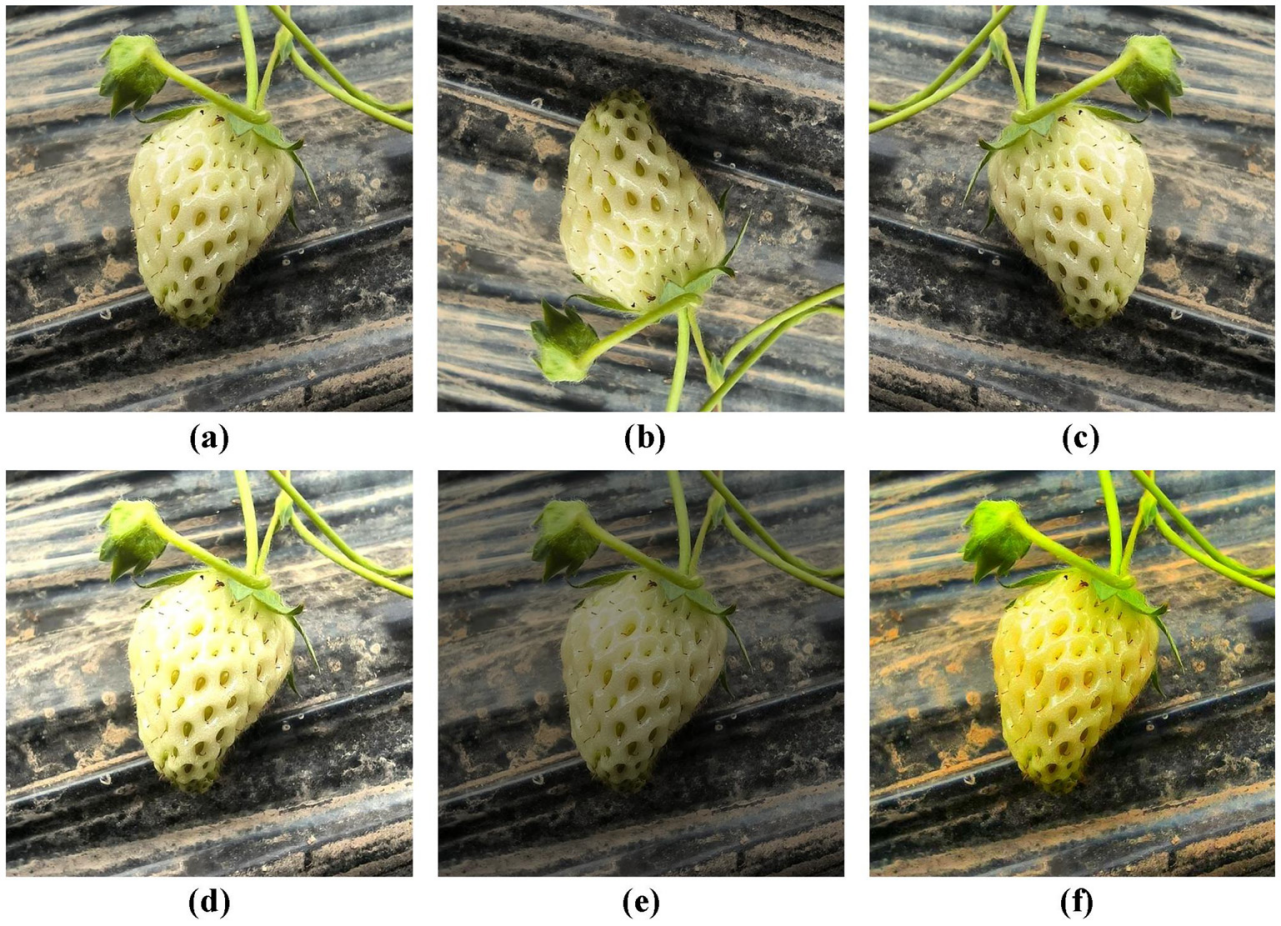
Image enhancement effects. **(A)** Original Image; **(B)** Image Flipping; **(C)** Image Mirroring; **(D)** Brightness Adjustment; **(E)** Darkness Adjustment; **(F)** Chroma Adjustment.

The data preprocessing methods employed in this paper significantly expand the scale and diversity of the strawberry growth stage dataset, providing more robust and effective data support for training deep learning models (Zhang et al., 2023c). Specifically, through a series of carefully designed operations, these preprocessing techniques increase the quantity of data and enhance its quality. **Figure 3** illustrates the improvements in images following preprocessing.

**Figure 4:**
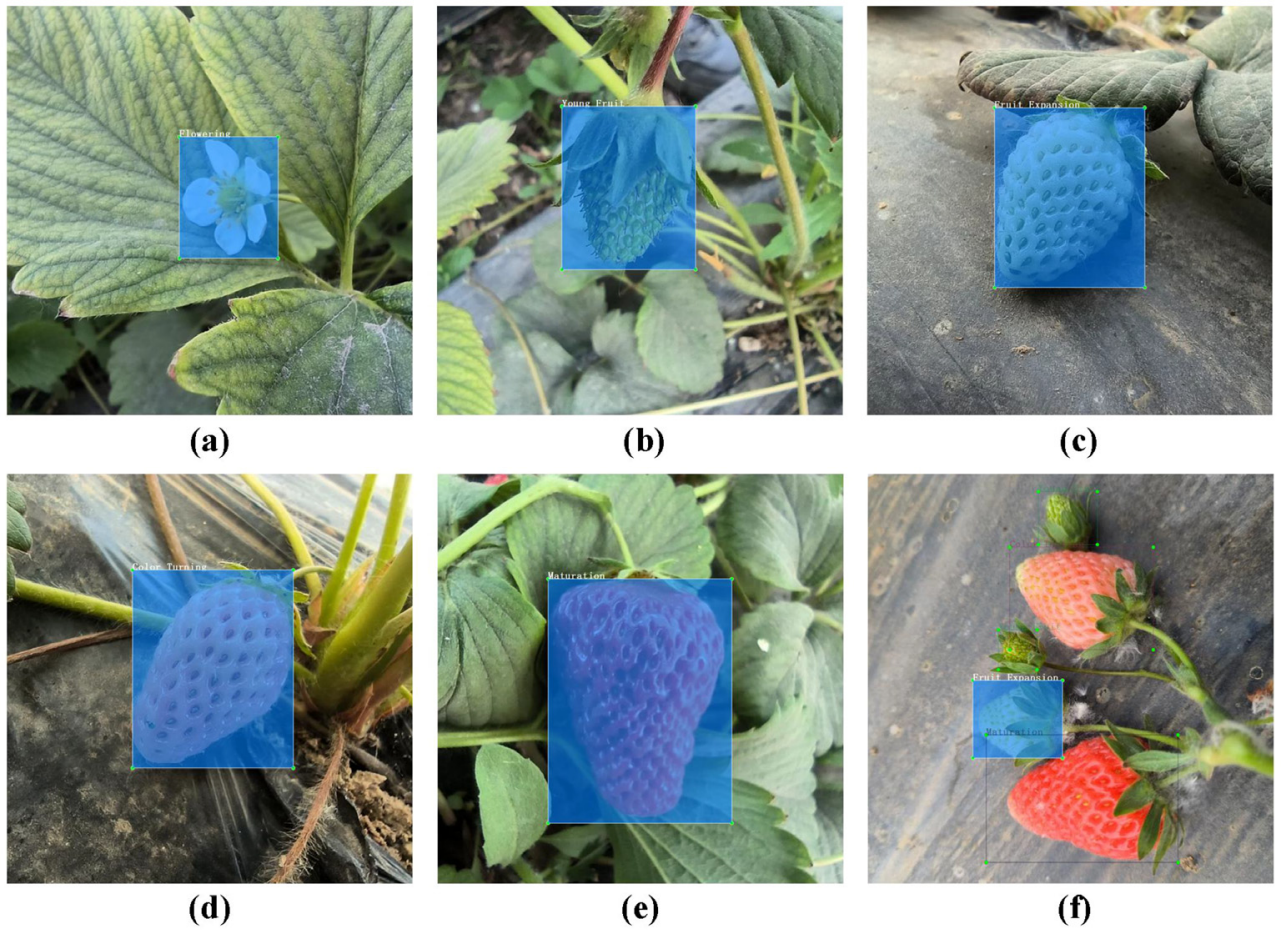
Different growth stages of strawberries. **(A)** Flowering; **(B)** Young Fruit; **(C)** Fruit Expansion; **(D)** Color Turning; **(E)** Maturation; **(F)** Multi-Stage Images.

To enhance the deep learning model’s ability to discern image details and improve its prediction accuracy, all images are uniformly resized to 640×640 pixels. This preprocessing step ensures consistency in model input and optimizes the efficiency of image feature extraction. To facilitate deep learning model training, the labels of the annotated data are saved in both PascalVOC and YOLO formats. **Figure 4** presents the data labeling results for each growth period.

After this adjustment, the dataset contains 7,528 images, with the training set comprising 6,022 images for model training and learning, while the test and validation set each contain 753 images for evaluating the model’s generalization ability and other performance metrics. **Table 2** provides a detailed breakdown of the dataset’s division and specific quantities.

**Table 2:** Data from different strawberry growth stages. “Raw Data” refers to the unprocessed data volume for each category, while “Processed Data” refers to the data after processing for each category.

Growth Stages	Original Data	Processed Data
Flowering	209	1331
Young Fruit	197	1293
Fruit Expansion	194	1289
Color Turning	192	1285
Maturation	206	1327
Multi-Stages	125	1003
Total	1123	7528

### 3.3 The Architecture of SGSNet

**Figure 5:**
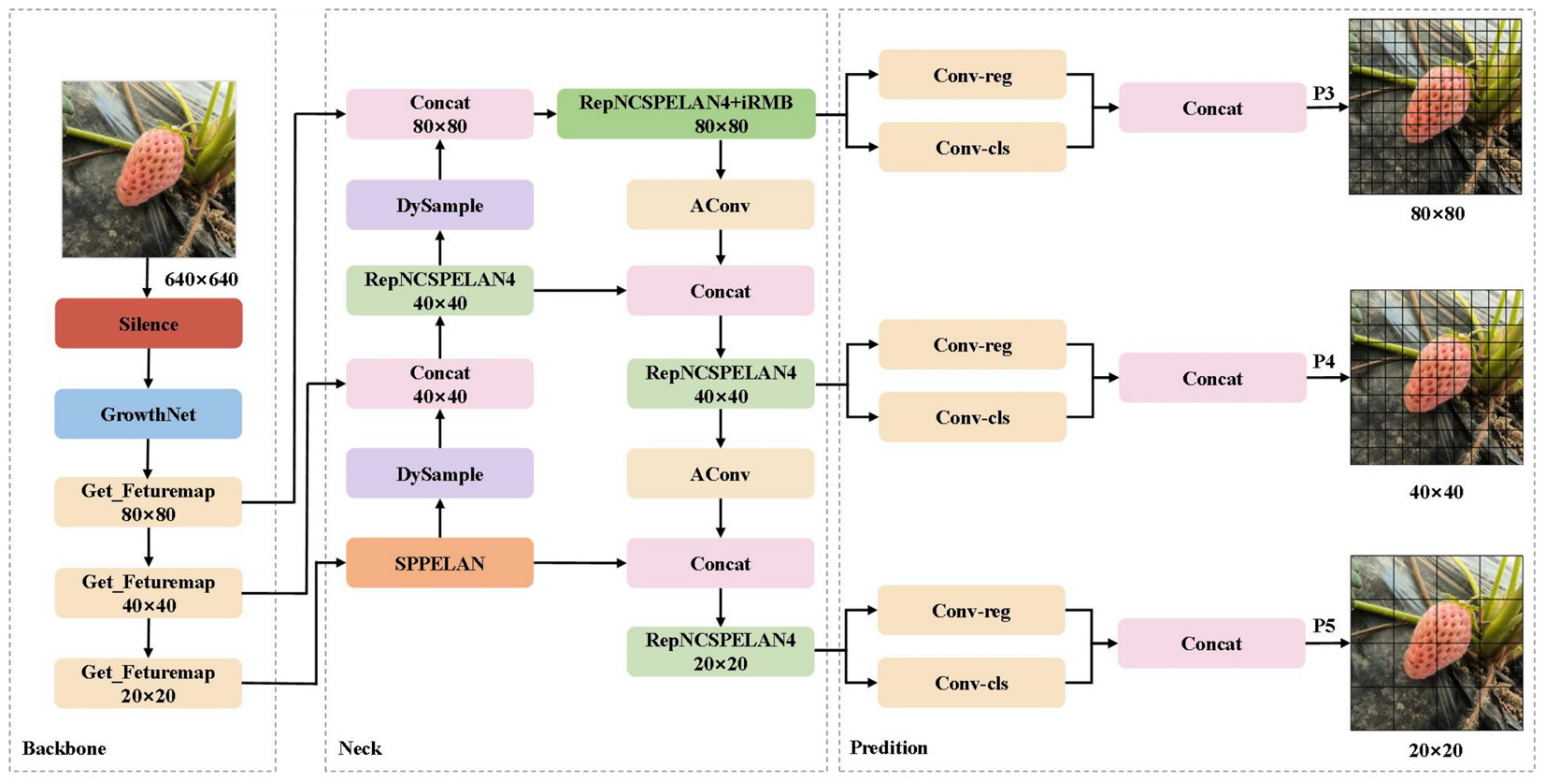
The architecture of SGSNet. The Backbone extracts primary features from input data. The Neck connects the Backbone to the Prediction module, processing and fusing features. The Prediction module handles target bounding box regression and feature map classification. The 640×640 denotes the initial image resolution, while 80×80, 40×40, and 20×20 indicate feature map resolutions for detecting targets at different scales.

The design of SGSNet draws on the structure of YOLOv9s (Liu et al., 2024b) but omits the Programmable Gradient Information (PGI) mechanism, replacing its backbone network GELAN with a self-developed, more lightweight GrowthNet. Despite incorporating deep feature fusion modules, YOLOv9s shows limitations in detecting small objects. SGSNet addresses this by integrating the DySample adaptive upsampling structure, which improves sensitivity to objects of various sizes, and by utilizing the iRMB module to enhance feature fusion, significantly improving the accuracy of small object detection. These improvements ensure that SGSNet meets the detection performance requirements across all growth stages of strawberries, as shown in **Figure 5**.

After data is entered into SGSNet, the Silence module pre-processes it, and the underlying features are extracted using the GrowthNet backbone network. These multi-level feature maps undergo processing through three fusion paths. The first path focuses on small-scale target detection, where the feature map is processed by adaptive upsampling through the DySample module (Liu et al., 2023). The RepNCSPELAN4 layer is then optimized using the iRMB attention mechanism (Zhang et al., 2023b) to enhance multi-scale feature fusion and improve the detection ability of small-scale targets. The second path targets mesoscale detection, where the feature map undergoes similar processing as in the first path and is further optimized through the AConv (Ma et al., 2021) layer before being merged by the Concat layer to enhance the model’s ability to detect mesoscale targets. The third path is dedicated to large-scale target detection, utilizing the SPPELAN layer for spatial pyramid pooling to improve the model’s capability in detecting large-scale targets. Finally, the outputs from the three paths are input into the Conv-reg and Conv-cls layers for bounding box regression and classification. The P3, P4, and P5 layers represent feature maps for small, medium, and large targets, respectively, enabling accurate detection of strawberries from flowering to ripening.

#### 3.3.1 The Get_Featuremap layer

The Get_Featuremap layer extracts feature maps from the GrowthNet network. After convolution operations, it produces multi-level feature maps. Each layer of these feature maps represents growth stage features at different scales, including low-level edge features and high-level semantic information. The feature extraction process of this module is illustrated in Equation 4.


(4) F(x)=Conv(W×x)

where *F*(*x*) denotes the feature map, where *x* represents the input image, *W* is the weight matrix of the convolution kernel, and *Conv* refers to the convolution operation.

#### 3.3.2 The RepNCSPELAN4 layer

The RepNCSPELAN4 layer integrates reparameterization techniques with multi-scale feature processing mechanisms to enhance multi-scale object detection capabilities. During the training phase, RepNCSPELAN4 employs a multi-branch feature fusion structure, enriching feature representations. In the inference phase, these branches are simplified into a single convolution operation through reparameterization, significantly improving inference speed and efficiency. Equation 5 illustrates the reparameterization formula.


(5) $$F_{Out}=Conv(W_{1}\times F_{1}+W_{2}\times F_{2}+\ldots +W_{n}\times F_{n})$$

where *F_1_, F_2_,…, F_n_* are the input feature maps from different branches, *W_1_, W_2_,…, W_n_* are the corresponding convolutional weights, and the final output feature map is *F_Out_*.

#### 3.3.3 The Concat layer

The Concat layer concatenates feature maps of different scales along the channel dimension, enabling SGSNet to integrate feature maps of various resolutions. This facilitates improved handling of multi-scale objects and ensures the model can simultaneously address detection tasks for large, medium, and small objects.

#### 3.3.4 The AConv layer

The Adaptive Convolution (AConv) layer is a lightweight convolutional module that enhances the nonlinear representation capabilities of convolutional networks through a dynamic activation mechanism, thereby improving the expressiveness of feature maps. Equation 6 illustrates the computational process of AConv.


(6) $$F_{AConv}=Conv(W\times F_{Concat})$$

where *W* represents the convolutional weights, *F_Concat_* denotes the concatenated feature map from the Concat module, and *F_AConv_* is the computed result.

#### 3.3.5 The SPPELAN layer

The SPPELAN layer is designed based on Spatial Pyramid Pooling, enabling pooling operations on feature maps at different scales. This design allows the model to capture richer contextual information across various scales, enhancing its robustness in detecting large and small targets. Equation 7 illustrates the pooling process of SPPELAN.


(7) $$F_{SPPELAN}=[Pool(F_{1},s_{1}),Pool(F_{2},s_{2}),\ldots ,Pool(F_{n},s_{n})]$$

where *F_SPPELAN_* represents the feature map after pooling, *Pool* denotes the pooling operation, *F_1_, F_2_,…, F_n_* are the results of pooling operations applied to the input feature maps, and *s_1_,s_2_,…, s_n_* represent different pooling scales.

#### 3.3.6 The DySample layer

DySample is an adaptive upsampling technique designed to meet the multi-scale feature extraction needs for detecting different stages of strawberry growth. As strawberries progress from blooming to ripening, their size and shape undergo significant changes, making traditional fixed upsampling methods inadequate for accurately capturing these dynamic features. In contrast, DySample offers a more efficient upsampling approach with its flexible, adaptive sampling mechanism, making it particularly suitable for complex tasks that require multi-scale object detection.

The primary advantage of DySample lies in its ability to dynamically adjust the sampling point positions, effectively overcoming the limitations of fixed kernel upsampling methods. By treating the upsampling process as a point-based resampling problem, DySample learns the offset for each pixel and constructs an adaptive set of sampling points, S. It then utilizes bilinear interpolation to perform precise resampling of the input feature map. This approach not only simplifies the complex convolutional calculations but also significantly enhances the accuracy and representative capacity of the upsampling process. The upsampling process of DySample is illustrated in Equation 8.


(8) x'=grid_sample(x,S)

where *x* denotes the input feature map, *S* represents the set of sampling points dynamically generated based on the offset *O*, and *x*′ indicates the resampled feature map.

DySample generates a new set of sampling points *S* by dynamically adjusting the offsets, with specific calculations detailed in Equation 9.


(9) S=G+O

where *G* represents the original set of sampling points, and *O* denotes the offsets generated by linear projection, which are ultimately used for resampling the feature map.

#### 3.3.7 The Conv-reg and Conv-cls layers

The Conv-reg layer is designed for regressing boundary box coordinate parameters, which precisely define the position and extent of target objects within an image. The Conv-cls layer accurately classifies each target object into its corresponding category, effectively differentiating diverse targets in complex scenes. The combination of these modules allows SGSNet to predict the precise location of target objects and determine their respective categories, thereby providing the final detection results.

### 3.4 The lightweight backbone network of GrowthNet

**Figure 6:**
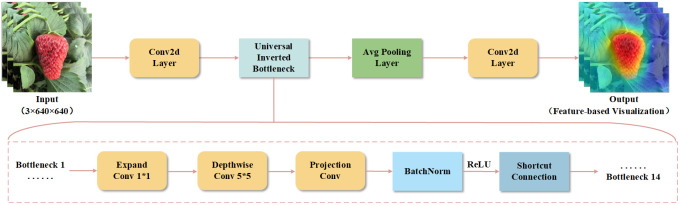
The architecture of GrowthNet. The input is a three-channel (RGB) image with a resolution of 640×640. The output is processed using feature visualization techniques to interpret the feature extraction outcomes of the model.

To enhance the lightweight characteristics and efficiency of SGSNet, a new architecture known as GrowthNet has been developed based on MobileNetv4 (Qin et al., 2024). MobileNetv4 introduces a Universal Inverted Bottleneck (UIB) that integrates multiple convolution strategies, thereby improving the network’s flexibility and efficiency. In contrast, GrowthNet further innovates by eliminating the fully connected layers found in the traditional MobileNetv4 framework, significantly simplifying the network structure and reducing computational overhead. This modification renders GrowthNet a more suitable backbone network for the precise detection tasks required across various growth stages of strawberries. Additionally, GrowthNet optimizes the interaction between batch normalization and ReLU activation layers, which stabilizes the training process and enhances the network’s robustness to variations in input data. These improvements are essential for maintaining high accuracy in real-time detection and classification tasks within SGSNet. The architectural design of GrowthNet not only fulfills the demands for efficient computation but also ensures optimal detection accuracy, making it particularly suitable for integration with deep learning models that require real-time processing and rapid inference capabilities, as illustrated in **Figure 6**.

After the 640×640 resolution image is input into GrowthNet, it undergoes feature extraction and processing. The data first passes through a 3×3 Conv2d layer for initial feature extraction and resolution reduction. The Universal Inverted Bottleneck Block (UIB) further compresses the image features, retaining core information while minimizing computational overhead. Following these bottleneck modules, the feature map is sent to the global average pooling layer, which aggregates the entire feature map into a low-dimensional feature vector for subsequent classification. The feature map is then refined through an additional Conv2d layer.

#### 3.4.1 The Conv2d layer

The 2d convolutional (Conv2d) layer applies a 3×3 convolutional kernel to the input image, reducing spatial resolution through a convolution operation with a stride of 2. This step extracts low-level features, such as edges and textures of strawberries at different growth stages, while decreasing computational complexity by reducing the resolution. Equation 10 illustrates the computation process.


(10) $$F_{Out}=Conv2D(F_{In},W,b)$$

where *F_In_* represents the input feature map, *W* denotes the convolutional kernel, *b* is the bias term, and *F_Out_* is the output feature map.

#### 3.4.2 The universal inverted bottleneck block layer

The Universal Inverted Bottleneck Block (UIB) is a core module of MobileNetv4. This module initially expands the dimensionality of the input feature map using an expansion convolution (Expand Conv). It then extracts spatial features independently from each channel using depthwise convolution. Finally, it projects the feature map to a lower dimension using pointwise convolution. The UIB structure is flexible and can be adjusted based on optimization objectives. Expanding the feature space reduces computational complexity through depthwise convolution, maintaining model efficiency. Equation 11 illustrates the feature processing process of this module.


(11) $$F_{Out}=PW(DepthwiseConv(ExpandConv(F_{In})))$$

where *F_In_* denotes the input feature map, *ExpandConv* refers to the expansion convolution operation, *DepthwiseConv* represents the depthwise convolution operation, *PW* indicates the pointwise convolution operation, and *F_Out_* signifies the output feature map.

#### 3.4.3 The depthwise Conv layer

Depthwise Convolution (Depthwise Conv) is a critical operation within the UIB module. It performs convolution independently on each input channel, significantly reducing computational complexity. Unlike standard convolution, depthwise convolution does not perform operations across channels but applies convolution separately to each channel. Equation 12 represents the depthwise convolution operation.


(12) $$y(i,j,c)=\sum_{m,n}x(i+m,j+n,c)\times w(m,n,c)$$

where *x* represents the input feature map, *w* denotes the convolutional kernel weights, *m* and *n* are the spatial position indices of the kernel, *y* (*i,j,c*) is the result of the convolution operation, and *c* indicates the channel index.

#### 3.4.4 The projection Conv layer

Projection Convolution (Projection Conv) projects the expanded feature maps back to the original dimensions, maintaining efficient computational performance. It enables the re-projection of high-dimensional features to lower dimensions, thus reducing computational requirements. The operation process is illustrated in Equation 13.


(13) $$F_{proj}=PW(F_{exp})$$

where *F_exp_* denotes the expanded high-dimensional feature map, *PW* represents the pointwise convolution operation, and *F_proj_* refers to the projected low-dimensional feature map.

#### 3.4.5 The BatchNorm layer

Batch Normalization (BatchNorm) is used to normalize the output of each layer to accelerate model training and enhance model stability. Normalizing each layer’s output to a consistent distribution prevents issues such as vanishing or exploding gradients. The normalization process is illustrated in Equation 14.


(14) $$y=\gamma \times \frac{x-\mu }{\sqrt{\sigma^{2}+\epsilon }}+\beta$$

where *x* represents the input feature map, *μ* and *σ* ^2^ denote the mean and variance of the batch, respectively, *ϵ* is a small constant to prevent division by zero, and *γ* and *β* are trainable scaling and shifting parameters. *y* represents the normalized output feature map.

#### 3.4.6 The ReLU activation function

Rectified Linear Unit (ReLU) activation function introduces non-linearity by truncating negative values to zero while retaining positive values. This approach enhances the model’s expressive power and effectively mitigates the vanishing gradient problem, thereby improving the training performance of neural networks. The operation process is illustrated in Equation 15.


(15) y=max(0,x)

where *x* represents the input features, and *y* denotes the output after ReLU activation.

#### 3.4.7 The shortcut connection layer

Shortcut Connection creates a shortcut path by directly adding the input features to the output, effectively bypassing some layers. This approach ensures that part of the initial input information is preserved even after undergoing expansion, depthwise, and projection convolution, enhancing the network’s learning capability and stability.

Average Pooling Layer (Avg Pooling Layer) reduces the dimensionality of feature maps by applying global average pooling to each channel, resulting in a 1x1 feature vector. This pooling process significantly decreases the feature dimensions while preserving the most critical global feature information. It reduces the risk of overfitting and enhances the model’s generalization capability.


(16) $$F_{Pool}=\frac{1}{N}\sum_{i=1}^{N}F_{In}(i)$$

where *F_In_* represents the input feature map, *N* denotes the size of the pooling window, and *F_pool_* refers to the output after pooling.

### 3.5 Feature fusion enhancement algorithm based on iRMB

The complex greenhouse environment limits the ability of image acquisition equipment to capture images of strawberries at various growth stages from close range. To address this challenge, a lightweight attention mechanism known as the Inverted Residual Mobile Block (iRMB) has been integrated into SGSNet to enhance the detection capabilities for small targets and improve multi-scale feature fusion. The iRMB is strategically positioned within the GrowthNet architecture, as this location effectively consolidates feature information from different scales, optimizing detection accuracy for small targets. Furthermore, the introduction of iRMB at this critical juncture allows the model to better adapt to the challenges posed by size variations of strawberries throughout their growth, thereby enhancing overall detection performance across all growth stages. This approach ensures that the model retains its lightweight characteristics while delivering exceptional detection performance throughout the strawberry growth process.

The core of the iRMB module lies in its use of Expanded Window Multi-Head Self-Attention (EW-MHSA) and Depthwise Convolution (DW-Conv) to enhance long-range dependency modeling and local feature extraction capabilities, respectively.


(17) $$F_{iRMB}(x)=DW-Conv(EW-MHSA(x))$$

where *F_iRMB_* (*x*) represents the feature map processed by the iRMB module, where xxx denotes the input feature map. *EW-MHSA* (*x*) is the output of the Expanded Window Multi-Head Self-Attention mechanism, which captures long-range dependencies across the entire feature map. *DW-Conv* (*EW-MHSA* (*x*)) applies depthwise convolution to the features after self-attention processing, enhancing the extraction of local information.

Combining the iRMB and RepNCSPELAN4 feature fusion modules involves developing a hierarchical dual-stream feature fusion approach. This method effectively integrates and optimizes these features, thereby enhancing the overall detection performance of the model. The dual-stream feature fusion method consists of three key components: feature weighting, feature dot product fusion, and multi-layer feature output.


(18) $$h_{Cov}\left(x\right)=\left(\sum_{i=1}^{m}\lambda_{i}h_{RepNCSPELAN4}^{i}\left(x\right)+1\right)+\left(\sum_{j=1}^{n}\mu_{j}h_{iRMB}^{j}\left(x\right)+1\right)$$

where *h_RepNCSPELAN4_* denotes RepNCSPELAN4, *h_iRMB_* represents the iRMB module, *λ* and *μ* are trainable weights, and *x* denotes the input feature map.

Weighted addition introduces trainable weights *λ* and *μ* to each element of the features. These weights are applied to the original output to amplify or attenuate the model’s recognition capabilities. The weighted outputs are then summed to produce a scalar value, which quantifies the effectiveness of the neural network model based on this weighted multiplication. This mechanism enhances the model’s sensitivity to crucial features while effectively suppressing irrelevant features and noise, optimizing the feature representation quality. Subsequently, the weighted feature maps are fed into the feature fusion module for deep integration, which aims to optimize the overall expressiveness and robustness of the features by integrating multi-source information. During the feature fusion phase, dot-product fusion techniques facilitate deep interaction between feature maps. This process involves performing dot-product operations on the weighted feature maps to generate a richer and more discriminative feature representation. This approach enhances the intrinsic relationships between features and improves the model’s recognition and understanding capabilities in complex and variable scenarios. Ultimately, the model generates more accurate and reliable bounding box predictions through the synergy of dot-product fusion and multi-layer feature interaction. The overall process of the feature fusion algorithm is detailed in **Algorithm 1**.

Algorithm 1 **Feature fusion algorithm.**

**1: Input:** 
F(x)
//Input feature map x
**2: for** (i=1;i<=n;i++) **do**:
**3:** 
$h_{RepNCSPELAN4}\left(x\right)\leftarrow \sigma_{RepNCSPELAN4}(F(x))$
//Extract RepNCSPELAN4 features
**4:** 
$h_{iRMB}\left(x\right)\leftarrow \sigma_{iRMB}(F(x))$
//Extract iRMB features
**5:** 
λ,μ←trainable weights() 
//Initialize trainable weights
**6:** 
$weight_{RepNCSPELAN4}\leftarrow \lambda \times h_{RepNCSPELAN4}\left(x\right)+1\;$
//Apply weight to RepNCSPELAN4 features
**7:** 
$weight_{iRMB}\leftarrow \mu \times h_{iRMB}\left(x\right)+1$
//Apply weight to iRMB features
**8:** 
$hcov(x)\leftarrow weight_{RepNCSPELAN4}+weight_{iRMB}$
//Combine weight features
**9: If** 
confidence threshold met(hcov(x))
 **then**:
**10:** 
$h_{cov}(x)\leftarrow finalfeature\;fusion(h_{cov}(x))$


**11:/**/Refine fused features using the final layer for output confidence
**12: else**

**13: **continue
**14: endif**

**15: end for**

**16: Output** 
$h_{cov}(x)$
//Output fused feature map

### 3.6 Loss function optimization based on Inner-IoU

**Figure 7:**
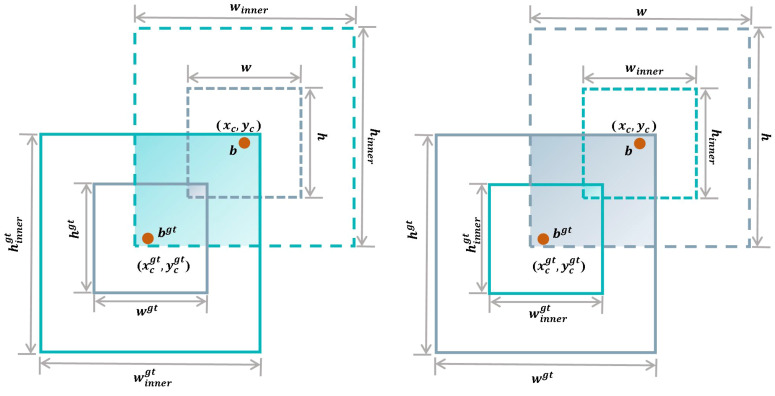
Description of Inner-IoU. The orange dots represent the centers of the predicted and ground-truth boxes. The dashed boxes denote the outer boundaries of the expected box *b* and the ground-truth box *b^gt^* in traditional IoU. The solid boxes illustrate the auxiliary boundaries *b_inner_* and $b_{inner}^{gt}$ introduced by the Inner-IoU.

Accurate bounding box regression is crucial for detecting strawberry growth stages. Although the traditional IoU loss function is widely used in such tasks, it exhibits slower convergence and limited generalization ability in specific contexts, especially when the overlap between predicted and ground-truth boxes is low or when significant scale differences exist. Given the considerable variation in the size of strawberries during growth, the loss function must possess more robust generalization capabilities. To address these issues, the Inner-IoU loss function (Zhang et al., 2023a) is introduced in the SGSNet. This approach markedly enhances the model’s regression performance, particularly in cases with substantial scale differences between predicted and ground-truth boxes, thereby improving convergence speed and detection accuracy. **Figure 7** illustrates the Inner-IoU loss function.

The inner-IoU loss function introduces the concept of auxiliary boundary boxes, with a scale factor serving as a tuning parameter to flexibly adjust the handling strategy for samples with varying IoU values. Equation 19 represents the inner boundary box of the ground-truth box *b^gt^*, and Equation 20 represents the inner boundary box of the predicted box $b_{inner}^{gt}$ .


(19) $$b_{gt}^{\text{inner}}=\left(x_{c}^{gt}-\frac{w_{gt}\times \text{ratio}}{2},y_{c}^{gt}-\frac{h_{gt}\times \text{ratio}}{2},w_{gt}\times \text{ratio},h_{gt}\times \text{ratio}\right)$$

(20) $$b^{\text{inner}}=\left(x_{c}-\frac{w\times \text{ratio}}{2},y_{c}-\frac{h\times \text{ratio}}{2},w\times \text{ratio},h\times \text{ratio}\right)$$

where *x_c_* and *y_c_* represent the center coordinates of the predicted and ground-truth boxes, respectively, while *w* and *h* denote the width and height, and the ratio is the scaling factor used to adjust the size of the inner boundary box.

After defining the inner boundary box, it is necessary to compute its intersection and union areas. Equations 21, 22 represent the calculation of the Intersection over Union (IoU) for the inner boundary box.


(21) $${\text{Inter}}_{\text{inner}}=\text{min}(b_{r},b_{gt_{r}})-\text{max}(b_{l},b_{gt_{l}})$$

(22) $${\text{Union}}_{\text{inner}}=w_{gt}^{\text{inner}}\times h_{gt}^{\text{inner}}+w^{\text{inner}}\times h^{\text{inner}}-{\text{inter}}_{\text{inner}}$$

where *b_r_* and *b_l_* represent the coordinates of the right and left boundaries of the bounding box, respectively.

After calculating the Intersection over the Union of the inner bounding boxes, the definition of the inner-IoU loss function can be obtained, as shown in Equation 23.


(23) $$L_{\text{Inner-IoU}}=1-\frac{{\text{inter}}_{\text{inner}}}{{\text{union}}_{\text{inner}}}$$

The introduction of the inner-ioU loss function demonstrates significant advantages for SGSNet in detecting different growth stages in strawberries. Specifically, this loss function accelerates the convergence process and improves regression accuracy when strawberry sizes are small. As strawberry sizes increase, auxiliary bounding boxes significantly reduce prediction errors, enhancing the model’s generalization capability and detection accuracy.

## 4 Experiments

### 4.1 Parameter setting

We utilized Python 3.9 and CUDA 11.6 within the PyCharm 2023 to build our model, operating on a 64-bit CentOS Linux 7 OS. The hardware setup included an Intel Xeon (R) Gold 6248R CPU and a Tesla V100S PCIE 32GB GPU. We extensively used the PyTorch framework, Torchvision library, and other image processing tools. This feature allowed for the real-time construction, modification, and debugging of our model, facilitating the development of a real-time strawberry growth period monitoring model. The specific parameter settings are detailed in **Table 3**.

**Table 3:** Parameters settings.

Parameter Name	Value	Parameter Name	Value
Momentum	0.937	Bounding Box Loss Factor	7.5
Initial Learning Rate	0.01	Classification Loss Factor	0.5
Final Learning Rate	0.01	Classification Loss Weight	1.0
Weight_Decay	0.0005	Objectness Loss Factor	0.7
Warmup_Epochs	3.0	Objectness Loss Weight	1.0
Warmup_Momentum	0.8	Dynamic Feature Loss Factor	1.5
Warmup_Learning Rate	0.1	Anchor Matching Threshold	5.0
Image Size	640×640	Optimizer	SGD
Batch Size	16	IoU Training Threshold	0.2

Model parameters have yet to converge in the initial stages of deep learning model training. Utilizing a high learning rate during this phase can result in substantial weight updates, potentially causing model instability. The warmup phase gradually increases the learning rate and momentum, smoothing the training process and effectively preventing the gradient explosion caused by high initial learning rates.

### 4.2 Evaluation indicators

The performance of SGSNet on the strawberry growth stages dataset is evaluated using metrics such as Recall, Precision, F1 score, Loss value, and mAP@0.5. “True positive” (*TP*) refers to the number of instances where actual positives are correctly identified. “False negative” (*FN*) indicates cases where positives are incorrectly classified as negatives. “False positive” (*FP*) denotes instances where negatives are wrongly identified as positives. “True negative” (*TN*) represents instances where negatives are accurately classified.

Precision is a metric used to measure the accuracy of a classification model, reflecting the percentage of samples correctly identified as positive out of all the samples predicted to be positive by the model, as illustrated in Equation 24:


(24) $$Precision=\frac{TP}{TP+FP}$$

Recall, called Sensitivity or True Positive Rate, is a performance metric used in classification models. It quantifies the proportion of actual positive cases correctly identified by the model, as described in Equation 25:


(25) $$Recall=\frac{TP}{TP+FN}$$

F1 Score is a metric that combines precision and recall, providing a comprehensive evaluation of performance. It calculates the harmonic mean of precision and recall, assessing both the model’s positive predictive value and its ability to accurately identify positive cases, as shown in Equation 26:


(26) $$F1=\frac{2Precision\times Recall}{Precision+Recall}$$

Loss primarily indicates the difference between the predicted outcomes and the actual outcomes, as shown in Equation 27:


(27) $$Loss=\frac{-1}{n}\times \sum \left(\alpha \times \log h_{\alpha \_hat}+(1-\alpha )\times \log(1-h_{\alpha \_hat})\right)$$

where denotes the total number of samples, $h_{\alpha \_hat}$ represents prediction for the positive class, α denotes the ground truth, and log is the natural logarithm function.

Mean Average Precision (*mAP*) is a comprehensive performance metric that evaluates classification models, especially in object detection tasks. The mAP@0.5 refers to the Intersection over the Union threshold, the overlap ratio between the predicted bounding box and the ground truth bounding box. It calculates the average precision across multiple classes and at different thresholds. The *mAP* value provides insight into the model’s accuracy in identifying positive samples while minimizing false positives, as described in Equation 28:


(28) $$mAP=\frac{1}{N}\sum_{i=1}^{N}AP_{i}$$

where *N* is the number of classes and *AP_i_* represents the average precision for the *i*-th class.

AP is the area under the PR curve, as shown in Equation 29:


(29) $$AP=\int_{0}^{1}P(R)dR$$

where *P* (*R*) represents Precision at a given Recall level *R*, with the integral evaluated from 0 to 1, indicating that AP measures Precision across all Recall levels from 0 to 1.

We utilize Params and GFLOPs to measure the complexity of the model. The calculation formulas for the other two evaluation metrics are shown in Equations 30, 32:


(30) $$Params=\sum (K_{h}\times K_{w}\times C_{in}\times C_{out})$$

(31) $$FlOPs=\sum (K_{h}\times K_{w}\times C_{in}\times C_{out}\times H\times W)$$

(32) $$GFlOPs=\frac{FlOPs}{10^{9}}$$

where Params refers to the total number of trainable parameters in the model, GFLOPs indicate the number of floating-point operations. *C_in_* and *C_out_* represent the number of input and output channels. *K_h_* and *K_w_* represent the width and height of the convolution kernel. *H* and *W* represent the height and width of the feature map.

### 4.3 Ablation experiment

In the same experimental environment, the performance of five mainstream convolutional neural networks as backbone networks is compared with that of SGSNet utilizing GrowthNet on the strawberry growth period dataset. These networks include RepViT (Wang et al., 2024a), an advanced lightweight Vision Transformer (ViT) variant developed in 2024, known for its excellent balance of performance and latency optimization; FasterNet (Chen et al., 2023), released in 2023, which enhances computational efficiency while maintaining high accuracy; EfficientNetv2 (Tan and Le, 2021), an efficient convolutional neural network employing compound scaling technology and a novel training method; Inceptionv4 (Szegedy et al., 2017), which integrates the Inception module with Residual connections, recognized for its multi-level feature extraction and superior accuracy; and MobileNetv4, the latest version of the lightweight convolutional neural network featuring the Universal Inverted Bottleneck. The parameters for each convolutional neural network are detailed in **Table 4**.

**Table 4:** Convolutional neural network parameters.

Network	Parameter	Value
RepVit	Conv Layers	16
	Conv Kernel	3×3, 5×5
	Feature Dimension	192, 224, 256, 384
	Depthwise Separable Layer	Present
FasterNet	Conv Layers	16
	Conv Kernel	3×3
	Feature Dimension	256, 512, 1024, 2048
	Depthwise Separable Layer	Present
EfficientNetv2	Conv Layers	24 (Conv3×3)
	Conv Kernel	3×3, 5×5
	Feature Dimension	24, 48, 64, 128, 160, 256
	Depthwise Separable Layer	Present
Inceptionv4	Conv Layers	64
	Conv Kernel	1×1, 3×3, 5×5
	Feature Dimension	1536, 1792, 2048
	Depthwise Separable Layer	None Present
MobileNetv4	Conv Layers	22
	Conv Kernel	3×3, 5×5
	Feature Dimension	160, 320, 640, 1280
	Depthwise Separable Layer	Present
GrowthNet	Conv Layers	16
	Conv Kernel	3×3, 5×5
	Feature Dimension	128, 256, 512, 1024
	Depthwise Separable Layer	Present

**Figure 8:**
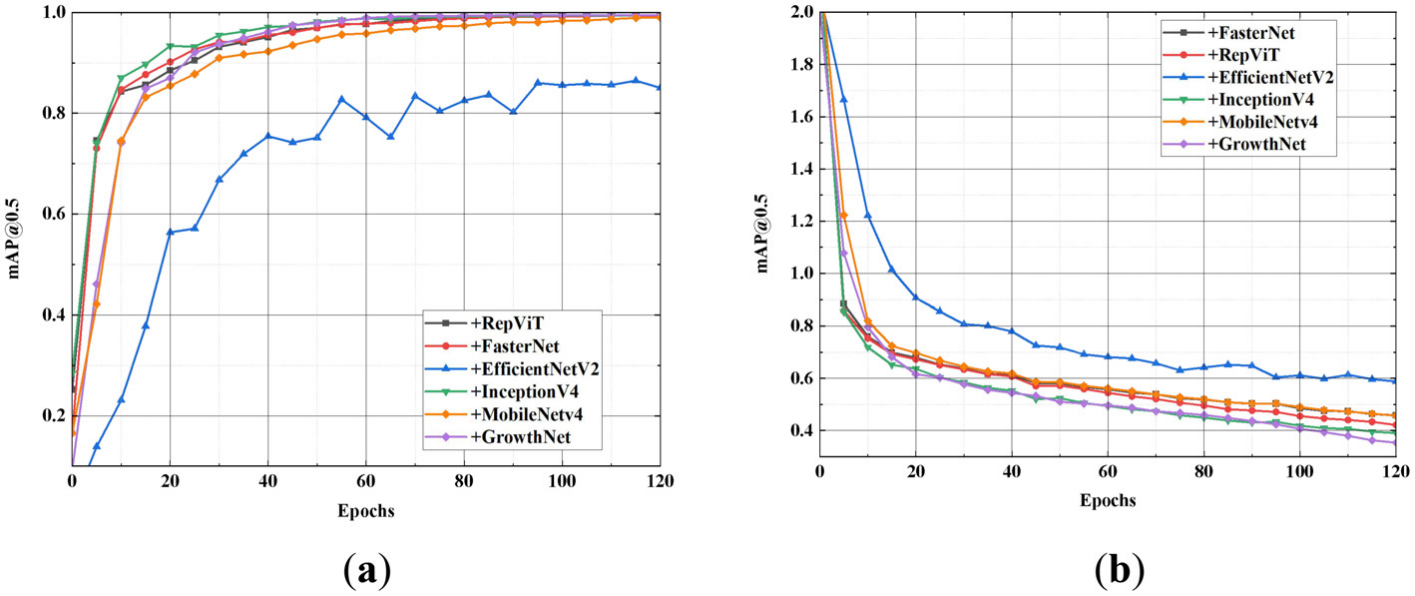
Comparison of mAP@0.5 rate and loss value. **(A)** mAP@0.5 rate; **(B)** Loss value.

The aforementioned convolutional networks serve as the backbone of SGSNet, and the performance advantages of SGSNet as a lightweight deep learning model are evaluated through a series of systematic and comprehensive comparisons. **Figure 8** presents the performance of different backbone networks within the model in terms of mAP and loss values.

As shown in **Figure 1**, the GrowthNet network, when used as the backbone of SGSNet, quickly achieves a higher mAP@0.5 value within the first 20 epochs and stabilizes thereafter. It demonstrates faster convergence and more stable high-precision performance, consistently maintaining a lower loss value and quicker convergence. The superior learning efficiency and robustness of SGSNet with the GrowthNet backbone are emphasized in comparison to other backbone networks configured for SGSNet. **Table 5** presents the performance of different backbone networks in the model across various comprehensive metrics.

**Table 5:** Comparison of different convolutional neural network settings for SGSNet backbone networks.

Method	Precision (%)	Recall (%)	F1 (%)	mAP@0.5 (%)	Loss	GFLOPs (B)
+RepVit	98.68	98.52	98.60	99.44	0.4216	18.4
+FasterNet	98.51	98.87	98.69	99.39	0.4586	17.3
+EfficientNetv2	82.48	78.52	80.45	85.03	0.5883	62.5
+Inceptionv4	98.25	98.78	98.51	99.45	0.3907	46.4
+MobileNetv4	98.53	99.07	98.80	98.96	0.4567	29.8
+GrowthNet	**98.83**	**99.45**	**99.14**	**99.50**	**0.3534**	**14.7**

The bold text represents the best values for each evaluation metric.

When GrowthNet is selected as the backbone network for SGSNet, it demonstrates superior performance in evaluation metrics such as precision, recall, F1 score, and mAP@0.5 compared to other backbone networks. SGSNet with GrowthNet achieves relatively low GFLOPs while maintaining accuracy and reliability. This capability enables the efficient deployment of SGSNet in resource-constrained environments, effectively balancing system performance and resource utilization efficiency.

**Figure 9:**
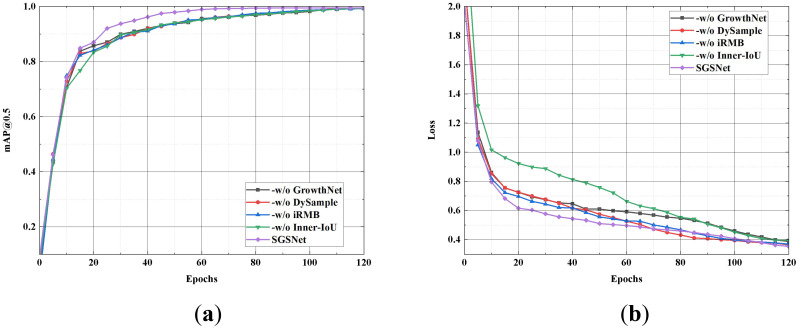
Comparison of mAP@0.5 rate and loss value. **(A)** mAP@0.5 rate; **(B)** Loss value.

To demonstrate the effectiveness of each module in SGSNet for detecting strawberry growth stages, module ablation experiments are conducted on the strawberry growth stages dataset. Specifically, the following ablations are performed on SGSNet: First, the original backbone structure of YOLOv9s is retained, while GrowthNet is removed (-w/o MobileNetV4). Second, the standard upsampling structure is maintained while DySample is removed (-w/o DySample). Next, the innovative feature fusion module iRMB in YOLOv9s is removed (-w/o iRMB). Finally, the improvement of the loss function combined with Inner-IoU is eliminated (-w/o Inner-IoU). **Figure 9** illustrates the performance of these ablations in terms of mAP@0.5 and loss values.

SGSNet achieves the highest mAP@0.5 and indicates superior performance. Removing GrowthNet slows the growth rate of mAP@0.5 and increases the loss value, highlighting GrowthNet’s importance in enhancing detection accuracy and efficiency. The removal of DySample results in a slight decrease in mAP and a slight increase in loss, indicating DySample’s role in maintaining high precision during the upsampling process. The absence of iRMB causes a significant drop in mAP and an increase in loss, demonstrating the critical contribution of iRMB to effective feature fusion. Finally, removing Inner-IoU decreases mAP and significantly increases loss, indicating its impact on optimizing the loss function and improving model reliability. **Table 6** presents the performance of each ablation operation across comprehensive metrics.

**Table 6:** Results of the ablation paper for each module.

Method	Precision (%)	Recall (%)	F1 (%)	mAP@0.5 (%)	Loss	GFLOPs (B)
-w/o GrowthNet	97.55	98.12	97.83	99.27	0.3873	28.6
-w/o DySample	98.01	97.84	97.92	99.31	0.3636	20.6
-w/o iRMB	98.34	98.08	98.21	99.29	0.3700	18.9
-w/o Inner-IoU	98.19	99.27	98.73	99.34	0.3965	14.7
SGSNet	98.83	99.45	99.14	99.50	0.3534	14.7

This table lists the best training results under different metrics.

The results indicate that SGSNet integrates all modules to achieve the best performance in precision, recall, F1 score, and mAP@0.5 while exhibiting the lowest loss and computational complexity. Removing GrowthNet significantly degrades performance, with precision dropping to 97.55% and loss increasing to 0.3873, highlighting its crucial role in enhancing detection accuracy and efficiency. The absence of DySample reduces precision to 98.01% and increases loss to 0.3636, underscoring its importance in maintaining high precision during the upsampling process. Excluding iRMB and Inner-IoU results in precision reductions to 98.34% and 98.19% and increases in loss to 0.3700 and 0.3965, respectively, demonstrating their critical contributions to effective feature fusion and loss optimization.

The ablation results demonstrate that integrating GrowthNet, DySample, iRMB, and Inner-IoU into SGSNet significantly enhances detection accuracy, efficiency, and reliability. This comprehensive integration effectively balances system performance and resource utilization, making SGSNet highly suitable for detecting strawberry growth stages in resource-constrained environments.

### 4.4 Generalization experiment

**Figure 10:**
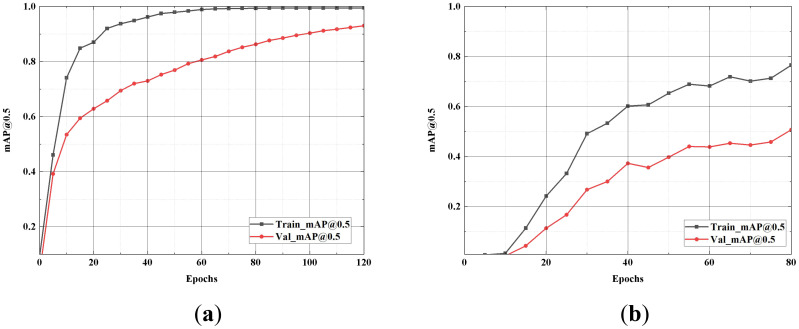
Comparison of accuracy rate. **(A)** presents the line graph of the model’s performance on the strawberry growth stages dataset after 120 iterations; **(B)** shows the line graph after 80 iterations on the self-constructed dataset. “Train_mAP@0.5” represents the training mAP@0.5, and “Val_mAP@0.5” represents the validation set mAP@0.5.

To evaluate the generalization capability of SGSNet, a comparative analysis of accuracy and loss rates is conducted across the training and testing sets of the strawberry growth stages dataset. This experiment utilizes a dataset consisting of 660 images, with each period containing 110 images. The training set, which comprises 80% of the dataset, includes 528 images sourced from a self-constructed dataset. The remaining 20%, consisting of 132 images, serves as the testing and validation set. The mAP@0.5 values for both the training and testing sets are illustrated in **Figure 10**.

**Figure 11:**
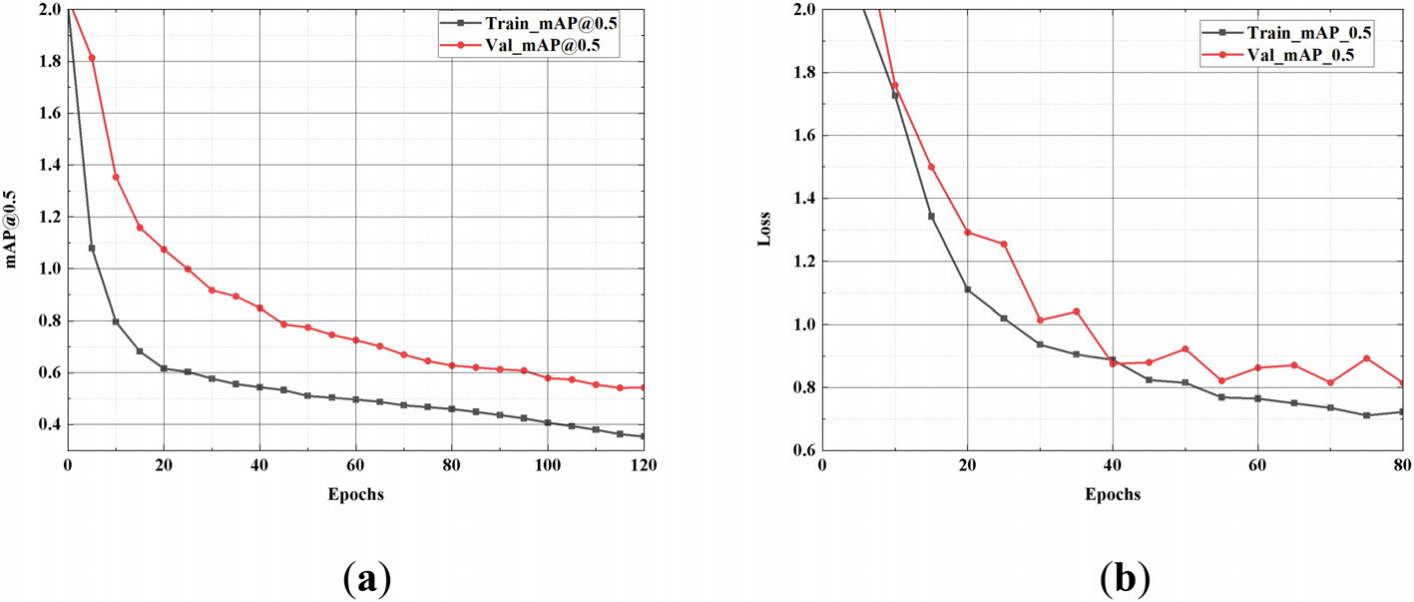
Comparison of loss value. **(A)** depicts the model’s performance on the strawberry growth stages after 120 iterations; **(B)** shows the line graph after 80 iterations on the self-constructed dataset. “Train-Loss” represents the training set Loss value, and “Val-Loss” represents the validation set Loss value.

The curves for the training and validation sets demonstrate that the model swiftly converges on the strawberry growth stages dataset with minimal fluctuations. In contrast, the curve for the dataset constructed for this experiment exhibits more pronounced volatility than the original dataset. Nevertheless, the mAP@0.5 of SGSNet increases with the number of training iterations on both datasets while the loss value decreases. The loss values for the training and testing sets are illustrated in **Figure 11**.

The results of the generalization experiments indicate that SGSNet demonstrates significant generalization capability across different datasets. The experiments involve datasets with distinct characteristics, including the strawberry growth stages dataset and a self-constructed dataset, to rigorously assess the model’s robustness. The model achieves efficient convergence, characterized by minimal fluctuations in mAP@0.5 and loss values, underscoring its effectiveness in learning from diverse data. This efficiency is particularly advantageous in scenarios with limited computational resources, as it enables SGSNet to maintain high accuracy while reducing computational complexity. Thus, SGSNet serves as an ideal solution for detecting and analyzing strawberry growth stages under various conditions.

### 4.5 Classification experiment

The improved Gradient-weighted Class Activation Mapping (Grad-CAM++) technology (Chattopadhay et al., 2018) is applied to provide better visual explanations for deep learning models. Based on existing Grad-CAM technology, Grad-CAM++ enhances object localization capability by introducing a pixel-level gradient weighting mechanism, especially in scenarios where multiple object instances are present in a single image. Grad-CAM++ calculates the feature map weights as shown in Equation 33:


(33) $$w_{k}^{c}=\sum_{i}\sum_{j}\alpha_{ij}^{kc}\times \text{ReLU}\left(\frac{\partial Y^{c}}{\partial A_{ij}^{k}}\right)$$

where $\alpha_{ij}^{kc}$ is the weight term used to adjust the gradient contribution, and $A_{ij}^{k}$ represents the value of the k -th feature map at the location in the final (i,j) convolutional feature map.

**Figure 12:**
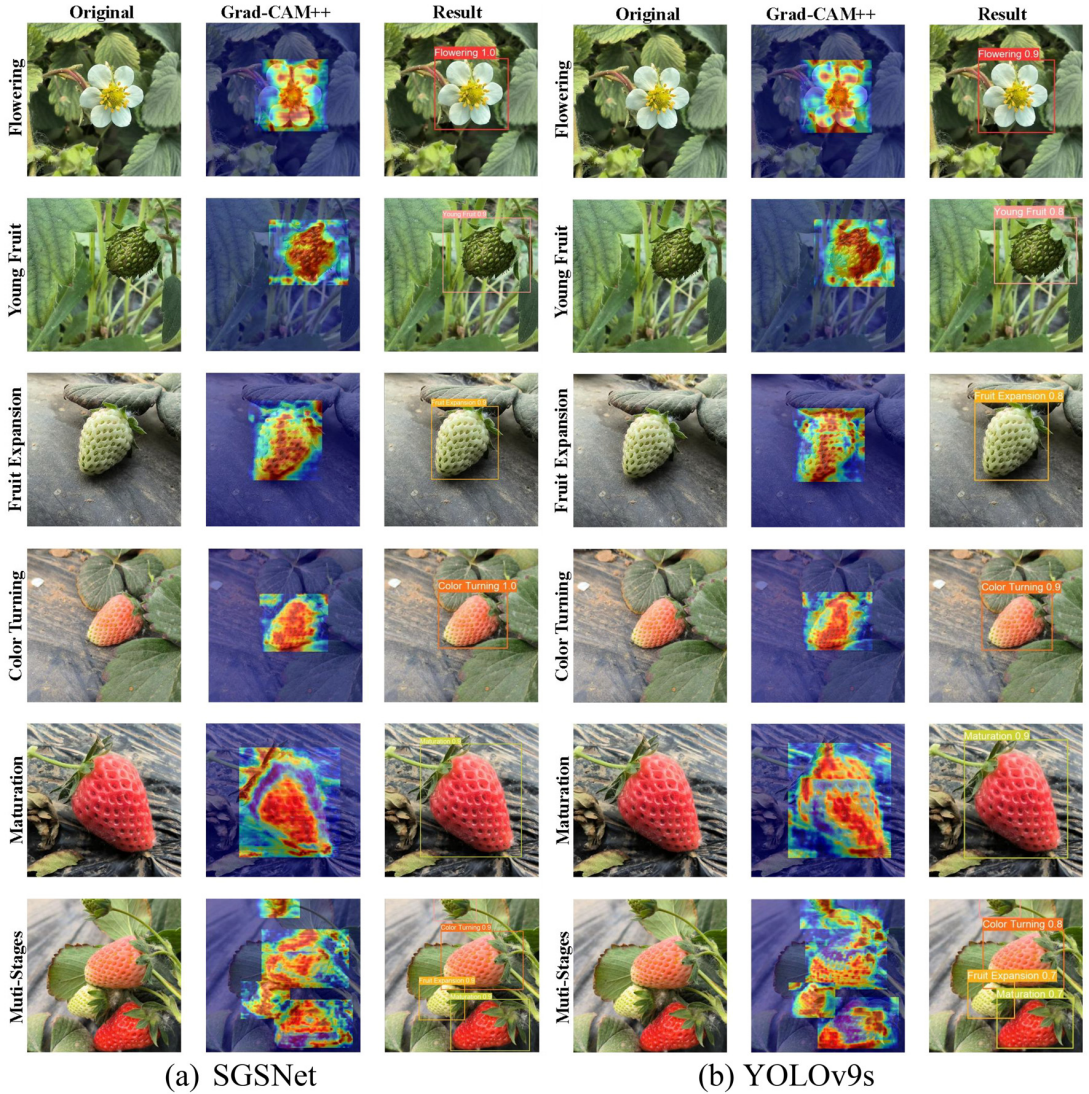
The visualizations of the strawberry growth stages using Grad-CAM++. These stages include flowering, young fruit, expansion, color turning, maturation, and multi-stage. Each row represents the original image, the Grad-CAM++ heatmap, and the model detection results.

Grad-CAM++ derives a closed-form solution for generating visual explanations through mathematical derivation and has been validated through extensive experiments on multiple tasks. The Grad-CAM++ heatmaps illustrate the model’s focus areas during feature extraction in the flowering, young fruit, fruit expansion, color turning, maturation, and multi-stage images. **Figure 12** illustrates the visualization results for the strawberry growth stages using Grad-CAM++.

The analysis of experimental results across various strawberry growth stages indicates that SGSNet exhibits superior localization capabilities in Grad-CAM++ heatmaps. Notably, during critical growth phases, the activated areas closely align with the actual target areas, reflecting a significant improvement in localization compared to YOLOv9s. In contrast, the activation areas of YOLOv9s are more dispersed, leading to reduced detection accuracy. Furthermore, SGSNet demonstrates higher confidence levels in detections across multiple stages; for instance, during the flowering and ripening phases, SGSNet achieves a confidence level of 1.0, whereas YOLOv9s presents comparatively lower confidence, suggesting that SGSNet is more reliable during the detection process, with a lower false positive rate. In addressing multi-scale targets, SGSNet effectively manages the varying scales of strawberries at different growth stages, particularly excelling in the detection of small and medium-sized targets within complex environments, while YOLOv9s exhibits relatively weaker performance in this area. In summary, SGSNet showcases enhanced precision, confidence, and multi-scale processing capabilities in detecting strawberry growth stages, thus offering a considerable advantage in precision agriculture applications. Comprehensive metrics of SGSNet performance across different growth stages are presented in **Table 7**.

**Table 7:** Classification experiment results. This table lists the best training results of the model at different growth stages under various metrics.

Stage	Precision (%)	Recall (%)	F1 (%)	mAP@0.5 (%)
Flowering	99.32	99.81	99.56	99.65
Young Fruit	98.31	94.52	96.38	99.32
Color Turning	98.94	99.67	99.30	99.49
Fruit Expansion	97.59	99.04	98.31	99.41
Maturation	99.43	99.78	99.60	99.63

### 4.6 Comparative experiment

The comparative experiments utilized the strawberry disease dataset to train widely recognized network models and the SGSNet. The extensive evaluation across various metrics highlighted the superior performance of the proposed model. The chosen versions of the YOLO series models are aligned with the YOLOv9s without auxiliary branches to ensure fairness in the comparative experiments. To achieve efficient and accurate comparisons across various growth phases of strawberries, the following models are selected: Faster R-CNN (Ren et al., 2016), YOLOv5s (Zhao et al., 2024a), YOLOv6s (Li et al., 2022a), YOLOv7 (Wang et al., 2023b), YOLOv8s (Wang et al., 2024e), YOLOv9s (Wang et al., 2024d), YOLOv10s (Wang et al., 2024b), RT-DERT-l (Zhao et al., 2024b), and RT-DERT-x.

With its two-stage process, Faster R-CNN excels in detecting overlapping targets in complex backgrounds but has a slower inference speed. The YOLO series models significantly enhance detection speed and accuracy with their single-stage design and improved feature extraction methods. Specifically, YOLOv5s improves feature extraction, greatly enhancing detection performance. YOLOv6s optimize network design and quantization techniques, increasing speed and accuracy and making it particularly suitable for industrial applications. YOLOv7 combines advanced network design and improved loss functions, performing exceptionally well in real-time detection tasks. YOLOv8s introduces the C2f module, further improving feature extraction and fusion capabilities. YOLOv9s uses CSP-ELAN blocks to achieve better gradient path planning and feature extraction, which are suitable for lightweight and large-scale models. YOLOv10s employs an efficiency-driven design and no-NMS training, significantly enhancing inference speed and parameter efficiency. The RT-DERT models balance real-time performance and global context understanding through the transformer architecture, further improving detection accuracy. Among them, RT-DERT-l focuses on extreme real-time performance, while RT-DERT-x balances accuracy and real-time performance. **Table 8** describes the parameter configurations of each comparative model.

**Table 8:** Comparison of network parameters.

Network	Parameter	Value
Faster R-CNN	Conv Layers	71
	Concat Layers	4
	Total Number Layers	78
	Feature Dimension	2048
YOLOv5s	Conv Layers	10
	Concat Layers	4
	Total Number Layers	24
	Feature Dimension	768
YOLOv6s	Conv Layers	23
	Concat Layers	4
	Total Number Layers	28
	Feature Dimension	256
YOLOv7	Conv Layers	77
	Concat Layers	15
	Total Number Layers	105
	Feature Dimension	1024
YOLOv8s	Conv Layers	7
	Concat Layers	4
	Total Number Layers	22
	Feature Dimension	512
YOLOv9s	Conv Layers	15
	Concat Layers	4
	Total Number Layers	22
	Feature Dimension	256
YOLOv10s	Conv Layers	15
	Concat Layers	4
	Total Number Layers	23
	Feature Dimension	512
RT-DERT-l	Conv Layers	14
	Concat Layers	4
	Total Number Layers	28
	Feature Dimension	256
RT-DERT-x	Conv Layers	16
	Concat Layers	4
	Total Number Layers	32
	Feature Dimension	384
SGSNet	Conv Layers	7
	Concat Layers	4
	Total Number Layers	19
	Feature Dimension	256

**Figure 13:**
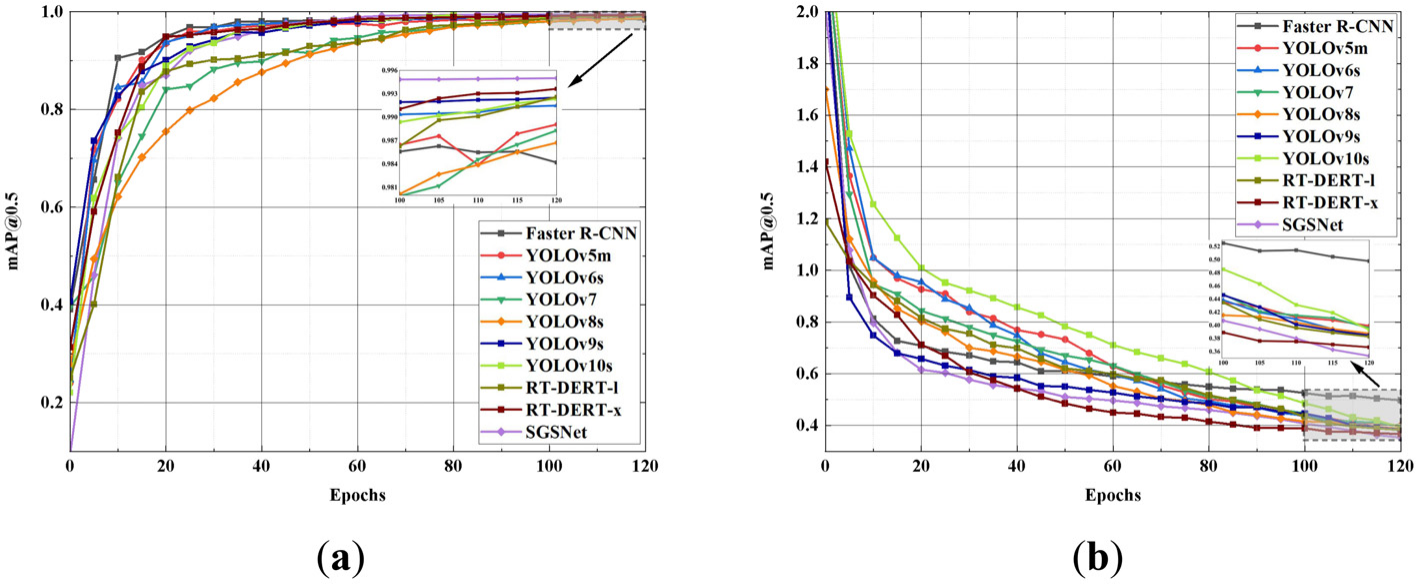
Comparison of mAP@0.5 rate and loss value. **(A)** mAP@0.5 rate; **(B)** Loss value.

To more intuitively observe the performance of SGSNet and the 10 comparative models in terms of mAP@0.5 and Loss values, **Figure 13** enlarges the plots of each model from 100 to 120 epochs.

**Figure 13** shows that within the first 20 epochs of training, SGSNet’s mAP@0.5 increases rapidly, surpassing most other models and demonstrating superior detection capabilities across various strawberry growth phases. In the early training stages, SGSNet achieves a rapid decrease in loss value, outperforming most comparative models and indicating strong learning ability and fast convergence in strawberry growth phase detection tasks. The enlarged plot from 100 to 120 epochs illustrates that SGSNet maintains relatively low and stable loss values, demonstrating model stability in later training stages. Compared to other models, SGSNet not only quickly increases mAP@0.5 and reduces loss values in the early training stages but also maintains good stability and efficiency in later stages, highlighting its competitiveness in strawberry growth phase detection tasks. **Table 9** presents the performance of the 10 comparative models across various comprehensive metrics.

**Table 9:** Comparison of different models.

Method	Precision (%)	Recall (%)	F1 (%)	mAP@0.5 (%)	Loss	GFLOPs (G)	Params (M)
Faster R-CNN	98.77	97.95	98.36	98.42	0.4978	251.4	41.3
YOLOv5s	97.69	95.83	96.75	98.91	0.3992	16.0	7.03
YOLOv6s	97.35	98.06	97.70	99.15	0.3864	44.2	16.31
YOLOv7	96.29	96.69	96.49	98.83	0.3959	105.2	37.22
YOLOv8s	97.46	95.75	96.60	98.67	0.3876	28.7	11.14
YOLOv9s	97.75	97.76	97.75	99.25	0.3848	26.9	7.20
YOLOv10s	97.81	96.78	97.29	99.24	0.3933	24.8	8.07
RT-DERT-l	98.17	97.63	97.90	99.26	0.3828	108.0	32.82
RT-DERT-x	98.23	98.11	98.17	99.36	0.3666	232.4	67.31
SGSNet	**98.83**	**99.45**	**99.14**	**99.50**	**0.3534**	**14.7**	**5.86**

The table presents the optimal training outcomes of the model across different metrics.

The bold text represents the best values for each evaluation metric.

The comparative data highlight the significant advantages of SGSNet in terms of parameter count and computational complexity. SGSNet has only 5.86 million parameters and 14.7 GFLOPs, which, although slightly higher than those of YOLOv5s, remain significantly lower than those of other models. SGSNet excels in lightweight design and surpasses all other comparative models in precision, recall, F1 score, and mAP@0.5. Additionally, it achieves an outstanding loss value of 0.3534. This difference underscores the ability of SGSNet to maintain high performance while achieving lower parameter counts and enhanced computational efficiency.

**Figure 14:**
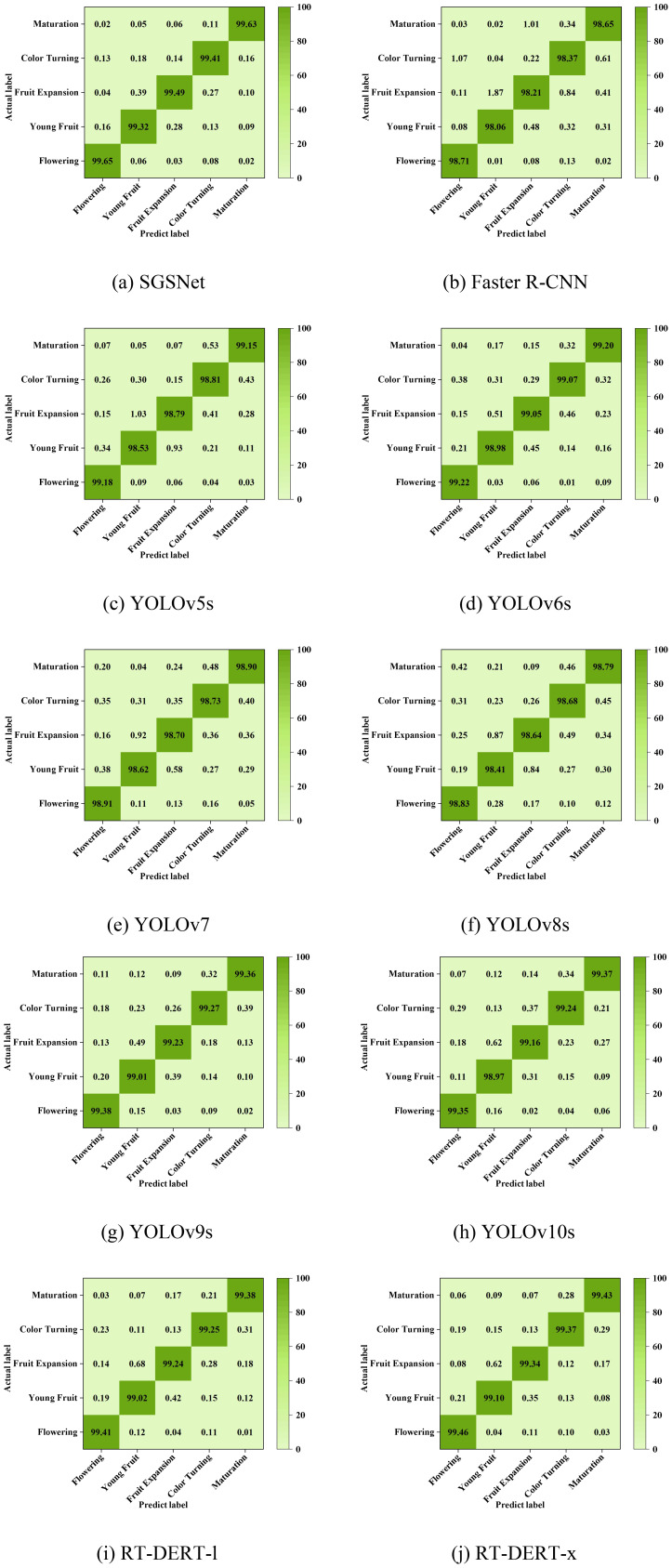
Confusion matrix of classification effect. **(A)** SGSNet; **(B)** Faster R-CNN; **(C)** YOLOv5m; **(D)** YOLOv6s; **(E)** YOLOv7; **(F)** YOLOv8s; **(G)** YOLOv9s; **(H)** YOLOv10s; **(I)** RT-DERT-l; **(J)** RT-DERT-x.

In summary, SGSNet stands out in detection tasks on datasets of different strawberry growth phases due to its high precision, low computational complexity, and exceptional overall performance. The confusion matrix in **Figure 14**, utilizing the mAP@0.5 metric, illustrates SGSNet’s performance in accurately identifying individual phases and compares it with Faster R-CNN, YOLOv5m, YOLOv6s, YOLOv7, YOLOv8s, YOLOv9s, YOLOv10s, RT-DERT-l, and RT-DERT-x.

The confusion matrix summarizes the recognition results, with rows representing predicted phases and columns representing actual phases. The diagonal elements indicate the probability of accurately detecting each strawberry growth phase, while the off-diagonal elements represent the likelihood of misclassification. The confusion matrix demonstrates that SGSNet achieves a higher mAP@0.5 in detecting various strawberry growth phases compared to other models. This observation strongly supports the effectiveness of SGSNet in detecting different strawberry growth phases.

## 5 Discussion

SGSNet is a lightweight deep learning model specifically designed for the accurate detection of strawberry growth stages, supported by a comprehensive dataset that covers all growth phases. It uses GrowthNet, an efficient lightweight convolutional neural network, as its backbone, significantly reducing model parameters and computational complexity. In contrast to traditional backbone networks, GrowthNet achieves a balance between efficiency and effectiveness, making SGSNet particularly suitable for deployment on resource-constrained devices such as drones or smartphones. The DySample adaptive upsampling structure dynamically adjusts sampling point positions, enabling optimized multi-scale detection. The RepNCSPELAN4 module is enhanced by the iRMB lightweight attention mechanism, which improves the accuracy of small target detection, while the loss function is refined with Inner-IoU to accelerate model convergence and enhance detection precision. Despite its impressive performance, SGSNet has limitations. While the model excels at detecting the growth stages of red-maturing strawberries, it faces challenges in detecting varieties that mature to white or light pink. Further validation is required to ensure effectiveness across different strawberry cultivars. Future research will focus on validating and refining the model for broader applicability across various strawberry cultivars.

## 6 Conclusion

This paper presents SGSNet, a lightweight deep learning model designed for efficient detection of strawberry growth stages. A comprehensive dataset covering various growth stages has been developed to provide a solid foundation for training and testing. SGSNet employs GrowthNet, a lightweight convolutional neural network, as its backbone, which significantly reduces the number of parameters and computational complexity while enabling efficient feature extraction and real-time processing. The model also incorporates the DySample adaptive upsampling structure, dynamically adjusting feature map resolution based on target size, greatly enhancing detection performance across strawberries of different sizes. The RepNCSPELAN4 module is optimized using the iRMB lightweight attention mechanism, facilitating efficient multi-scale feature fusion and improving precision in small target detection, particularly for long-distance images. Additionally, the loss function is refined with Inner-IoU, accelerating model convergence and further enhancing detection precision.

Testing results highlight the superior performance of SGSNet, achieving 98.83% precision, 99.45% recall, 99.14% F1 score, 99.50% mAP@0.5, and a loss value of 0.3534. SGSNet surpasses widely used models such as Faster R-CNN, YOLOv10, and RT-DERT-l. Additionally, SGSNet requires only 14.7 GFLOPs and 5.86 million parameters, balancing high performance with resource efficiency. Comprehensive experimental validation demonstrates that SGSNet outperforms current mainstream detection methods across various quantitative and qualitative evaluation metrics. The model delivers high detection accuracy while significantly optimizing inference speed and reducing parameter count, making it ideal for deployment in resource-constrained environments. In the future, applying SGSNet to other crops or agricultural products could pave the way for broader development of intelligent agricultural management systems. These systems could address challenges such as pest detection, growth monitoring, and yield estimation, contributing to advancements in precision agriculture.

## Data Availability

The raw data supporting the conclusions of this article will be made available by the authors, without undue reservation.
